# Associations of common polymorphisms in *GCKR *with type 2 diabetes and related traits in a Han Chinese population: a case-control study

**DOI:** 10.1186/1471-2350-12-66

**Published:** 2011-05-13

**Authors:** Yan Ling, Xiaomu Li, Qian Gu, Hongyan Chen, Daru Lu, Xin Gao

**Affiliations:** 1 Department of Endocrinology and Metabolism, Zhongshan Hospital, Fudan University, Shanghai 200032, China; 2 Department of Geriatrics, Zhongshan Hospital, Fudan University, Shanghai 200032, China; 3 The State Key Laboratory of Genetic Engineering and Key Laboratory of Contemporary Anthropology, School of Life Sciences, Fudan University, Shanghai 200433, China

## Abstract

**Background:**

Several studies have shown that variants in the glucokinase regulatory protein gene (*GCKR*) were associated with type 2 diabetes and dyslipidemia. The purpose of this study was to examine whether tag single nucleotide polymorphisms (SNPs) in the *GCKR *region were associated with type 2 diabetes and related traits in a Han Chinese population and to identify the potential mechanisms underlying these associations.

**Methods:**

We investigated the association of polymorphisms in the *GCKR *gene with type 2 diabetes by employing a case-control study design (1118 cases and 1161 controls). Four tag SNPs (rs8179206, rs2293572, rs3817588 and rs780094) with pairwise r^2 ^> 0.8 and minor allele frequency > 0.05 across the *GCKR *gene and its flanking regions were studied and haplotypes were constructed. Genotyping was performed by matrix-assisted laser desorption/ionization time-of-flight mass spectroscopy using a MassARRAY platform.

**Results:**

The G alleles of *GCKR *rs3817588 and rs780094 were associated with an increased risk of type 2 diabetes after adjustment for year of birth, sex and BMI (OR = 1.24, 95% CI 1.08-1.43, p = 0.002 and OR = 1.22, 95% CI 1.07-1.38, p = 0.002, respectively). In the non-diabetic controls, the GG carriers of rs3817588 and rs780094 were nominally associated with a lower plasma triglyceride level compared to the AA carriers after adjustment for year of birth, sex and BMI (p for trend = 0.00004 and 0.03, respectively). Furthermore, the association of rs3817588 with plasma triglyceride level was still significant after correcting for multiple testing.

**Conclusions:**

The rs3817588 A/G polymorphism of the *GCKR *gene was associated with type 2 diabetes and plasma triglyceride level in the Han Chinese population.

## Background

Glucokinase (GCK) is the key glucose phosphorylation enzyme responsible for the first rate-limiting step in the glycolysis pathway. GCK regulates glucose metabolism in the liver and glucose-stimulated insulin secretion from pancreatic beta cells [1]. GCK activity is closely regulated by the glucokinase regulatory protein (GCKR), a process depending on fructose 6-phosphate and fructose 1-phosphate [2,3]. *Gckr*-deficient mice display reduced GCK protein levels and activity in the liver and exhibit impaired postprandial glycemic control [4,5]. In a previous study, adenoviral-mediated hepatic overexpression of GCKR significantly improved insulin sensitivity and glucose tolerance in mice and resulted in decreased leptin concentration and increased triglyceride levels [6].

In the Diabetes Genetics Initiative genome-wide association study, the *GCKR *rs780094 A allele was found to be strongly associated with hypertriglyceridemia in populations from Finland and Sweden [7]. Subsequently, a large study of Danish white participants confirmed that the rs780094 A allele was associated with increased fasting triglycerides, impaired fasting and OGTT-related insulin release, reduced homeostasis model assessment of insulin resistance (HOMA-IR), increased risk of dyslipidemia and a modestly decreased risk of type 2 diabetes [8]. The HapMap II CEU data http://www.hapmap.org showed that rs780094 was in strong linkage disequilibrium (LD) (r^2 ^= 0.932) with a non-synonymous *GCKR *variant (Pro446Leu, rs1260326). The DESIR prospective cohort study demonstrated that the *GCKR *variant rs1260326 T allele was strongly associated with increased triglyceride levels, lower fasting glucose and insulin levels, a lower HOMA-IR index, and a higher risk for dyslipidemia, but a lower risk for hyperglycemia and type 2 diabetes in a general French population [9]. Another study, combining data from 12 independent cohorts comprising more than 45,000 individuals with various ethnic backgrounds, confirmed that *GCKR *rs780094 and rs1260326 were strongly associated with opposite effects on fasting plasma triglyceride and glucose concentrations [10]. Recently, the MAGIC study conducted a large-scale meta-analysis and provided convincing evidence that the *GCKR *rs780094 A allele was associated with lower fasting glucose and insulin levels, a lower HOMA-IR index, a higher triglyceride level, and a lower risk for type 2 diabetes [11].

Several studies of the association of *GCKR *variants with type 2 diabetes or glucose homeostasis parameters in Chinese populations have been reported [12-14]. In a study of a population-based sample of Han Chinese individuals, the *GCKR *rs780094 A allele was found to be significantly associated with a reduced risk of impaired fasting glucose (IFG) and type 2 diabetes, decreased fasting glucose, increased homeostasis model assessment of beta cell function (HOMA-B), and fasting triglyceride levels; *GCKR *rs1260326 displayed similar associations [12]. A study of healthy Chinese adults and adolescents showed that the *GCKR *rs780094 A allele was associated with increased triglyceride levels, and *GCKR *rs780094 alone did not contribute to fasting glucose but interacted with *GCK *rs1799884 to increase fasting glucose [13]. However, another study in a Han Chinese cohort did not find any association between *GCKR *rs780094 and type 2 diabetes [14]. Therefore, the association of *GCKR *variants with fasting plasma glucose and type 2 diabetes is still not confirmed in a Chinese population. The aim of this study was to replicate the associations of *GCKR *variants with type 2 diabetes and related traits found in Caucasian populations in a Han Chinese population and to identify the potential mechanisms underlying these associations.

## Methods

### Study population

All participants were of Southern Han Chinese ancestry and resided in the Shanghai metropolitan area. We recruited 1118 unrelated type 2 diabetic inpatients from the Endocrinology and Metabolism Department of Zhongshan Hospital, Fudan University, Shanghai, China. All diabetic patients met the 1999 WHO criteria for diabetes [15], had been diagnosed after the age of 29 years, and were treated with oral hypoglycemic agents and/or insulin. The 1161 unrelated non-diabetic control participants were recruited from people undergoing health examinations in Zhongshan Hospital, were older than 40 years, and had a fasting plasma glucose < 5.6 mmol/l.

Written informed consent was obtained from all participants and the study was approved by the ethnic committee of Zhongshan Hospital, Fudan University, Shanghai, China.

### Clinical measurements

Both the diabetic patients and the controls were extensively phenotyped for anthropometric and biochemical traits related to glucose metabolism. The phenotypes assessed in our study include height, weight, waist circumference, blood pressure, fasting glucose, total cholesterol, triglyceride, high density lipoprotein cholesterol (HDL-C), and low density lipoprotein cholesterol (LDL-C). BMI was calculated as weight (kg)/height^2 ^(m^2^). In a subgroup of diabetic patients (n = 664), potential beta cell function was determined using intravenous arginine stimulation tests under fasting conditions. After taking a baseline blood sample, a 10% (wt/vol.) solution of arginine hydrochloride (5 g) was injected intravenously for 30-45 s. The end of the injection period was designated as time zero, after which samples were taken at 2, 4 and 6 min. The acute insulin response (AIR) to arginine was calculated as the mean of the insulin levels in the postinjection samples minus the insulin level in the prestimulus sample. The acute C-peptide response (ACPR) to arginine was calculated in the same way using sampled C-peptide levels.

### Genotyping

We selected tag single nucleotide polymorphisms (SNPs) across the region of the *GCKR *gene (include 20 kb upstream and 9 kb downstream of the gene) from the HapMap Phase II, using the pairwise tagging model.

The pairwise tagging algorithm was developed by Carlson et al. and has been described previously [16]. In brief, the algorithm is based on the r^2 ^LD statistic and is comprised of several steps [16]. Starting with all SNPs above the specified minor allele frequency (MAF) threshold in the candidate gene region, the single SNP exceeding the specified r^2 ^threshold with the maximum number of other SNPs above the MAF threshold is identified [16]. This maximally informative SNP and all associated SNPs are grouped as a bin of associated sites [16]. Any SNP exceeding the threshold r^2 ^with all other SNPs in the bin is specified as a tag SNP for the bin [16]. Thus, one or more SNPs within a bin are specified as "tag SNPs" and only one tag SNP would need to be genotyped per bin. The binning process is iterated, analyzing all as-yet-unbinned SNPs at each round, until all sites exceeding the MAF threshold are binned [16]. Thus, the maximally informative set of common SNPs (tag SNPs) is selected and is to be assayed in candidate gene association studies [16]. All polymorphisms above a specified frequency threshold either are directly assayed or exceed a specified threshold level of r^2 ^with an assayed polymorphism (tag SNP) [16].

The selection criteria used in our study were an r^2 ^> 0.8 and a minor allele frequency > 0.05. Finally, four tag SNPs (rs8179206, rs2293572, rs3817588 and rs780094) were selected and genotyped. The genotyping was performed by matrix-assisted laser desorption/ionization time-of-flight mass spectroscopy using a MassARRAY platform (MassARRAY Compact Analyzer, Sequenom, San Diego, CA, USA).

### Statistical analysis

Continuous variables are expressed as the means ± SEM. Comparisons between groups were performed with T testing and χ^2 ^testing for normally distributed continuous and categorical variables, respectively. Deviations from the Hardy-Weinberg equilibrium were assessed by means of χ^2 ^testing. SNPs that were not in Hardy-Weinberg equilibrium were excluded from further analysis. Pairwise linkage disequilibrium including D' and r^2 ^was estimated using Haploview. Haplotypes estimating from the population genotype data were performed in Haplo. Stats (R2.8.1). We did allelic analysis for the association of *GCKR *polymorphisms with type 2 diabetes using logistic regression. We did genotypic analysis for the association of *GCKR *polymorphisms with quantitative traits using a general linear model, assuming an additive model. We tested the association of haplotypes with type 2 diabetes and quantitative traits by using logistic regression and a general linear model. Non-normally distributed values were log-transformed before analysis. All models were adjusted for year of birth and sex. Additional models were adjusted for BMI. Analysis was performed using SPSS software version 13.0. We used Bonferroni correction for multiple testing.

## Results

### Baseline characteristics

The baseline characteristics of participants in this study are presented in Table 1. Of 2279 participants, 1118 were type 2 diabetes patients and 1161 were non-diabetic controls. Diabetic cases were older and had higher BMI, waist circumference, fasting glucose and triglyceride levels, but lower cholesterol concentrations than non-diabetic controls. There was no significant difference in the distribution of sex between diabetic cases and non-diabetic controls (p = 0.34).

**Table 1:** Baseline characteristics of all genotyped participants, diabetic cases and non-diabetic controls

Characteristic	All participants (n = 2279)	Diabetic cases (n = 1118)	Non-diabetic controls (n = 1161)	P value*
Age (years)	58.3 ± 0.24	60.2 ± 0.37	56.5 ± 0.32	< 0.001
Men (%)	43.6	44.6	42.7	0.34
BMI (kg/m^2^)	24.0 ± 0.07	24.4 ± 0.11	23.5 ± 0.09	< 0.001
Waist circumference (cm)	85.6 ± 0.45	90.5 ± 0.81	80.5 ± 0.28	< 0.001
Fasting glucose (mmol/l)	6.30 ± 0.01	8.17 ± 0.04	4.86 ± 0.01	< 0.001
Total cholesterol (mmol/l)**^+^**	4.76 ± 0.03	4.43 ± 0.04	5.10 ± 0.03	< 0.001
Triglyceride (mmol/l)**^+^**	1.54 ± 0.02	1.64 ± 0.03	1.46 ± 0.02	< 0.001
HDL-C (mmol/l)**^+^**	1.24 ± 0.01	1.16 ± 0.01	1.32 ± 0.01	< 0.001
LDL-C (mmol/l)**^+^**	2.69 ± 0.02	2.40 ± 0.03	3.00 ± 0.02	< 0.001
Systolic BP (mmol/l)	126.8 ± 0.38	134.2 ± 0.53	119.4 ± 0.45	< 0.001
Diastolic BP (mmol/l)	79.4 ± 0.19	80.7 ± 0.28	78.0 ± 0.26	< 0.001

Continuous data are expressed as the means ± SEM

**^+^**Variables were log-transformed before statistical analysis; numbers in the table were back-transformed

*p value for comparison between cases and controls

### Associations of *GCKR *polymorphisms with type 2 diabetes

Overall, four SNPs (rs8179206, rs2293572, rs3817588 and rs780094) were selected and genotyped in the present study. The call rates of rs8179206, rs2293572, rs3817588 and rs780094 were 98.5%, 98.0%, 96.6% and 98.5%, respectively. The concordant rates of all SNPs based on 120 duplicates were 100%.

Rs8179206, rs2293572, rs3817588 and rs780094 were in Hardy-Weinberg equilibrium in the total population, diabetic cases and non-diabetic controls (Table 2). The G allele of rs3817588 was significantly associated with an increased risk of type 2 diabetes after adjustment for year of birth and sex (OR = 1.21, 95% CI 1.06-1.39, p = 0.004) (Table 3). In addition, the G allele of rs780094 was significantly associated with an increased risk of type 2 diabetes after adjustment for year of birth and sex (OR = 1.19, 95% CI 1.05-1.34, p = 0.005) (Table 3). The associations remained significant after additional adjustment for BMI (Table 3). The associations of rs8179206 and rs2293572 with type 2 diabetes were not significant (OR = 1.18, 95% CI 0.85-1.65, p = 0.32 and OR = 1.04, 95% CI 0.88-1.23, p = 0.64, respectively) (Table 3).

**Table 2:** Characteristics of SNPs genotyped in *GCKR*

	SNP identification	Chromosome Position	Relation to the gene	Major allele	Minor allele	MAF	HWE P value
1	rs8179206	22573946	Exon	A	G	0.03	0.76
2	rs2293572	27582281	Intron	G	C	0.14	0.85
3	rs3817588	27584716	Intron	A	G	0.30	0.10
4	rs780094	27594741	Intron	A	G	0.45	0.90

**Table 3:** Allelic distribution of *GCKR *polymorphisms and association with type 2 diabetes

Alleles	Non-DM (%)	DM (%)	Odds Ratio1^a^	95% CI	P value	Odds Ratio2^b^	95% CI	P value
rs8179206								
A	97.0	96.6	1	(Ref.)		1	(Ref.)	
G	3.0	3.4	1.18	0.85-1.65	0.32	1.29	0.91-1.82	0.15
rs2293572								
G	85.9	85.6	1	(Ref.)		1	(Ref.)	
C	14.1	14.4	1.04	0.88-1.23	0.64	1.04	0.88-1.24	0.64
rs3817588								
A	72.1	68.0	1	(Ref.)		1	(Ref.)	
G	27.9	32.0	1.21	1.06-1.39	0.004*	1.24	1.08-1.43	0.002*
rs780094								
A	56.6	52.6	1	(Ref.)		1	(Ref.)	
G	43.4	47.4	1.19	1.05-1.34	0.005*	1.22	1.07-1.38	0.002*

^a ^Adjusted for year of birth and sex

^b ^Adjusted for year of birth, sex and BMI

*Significant after Bonferroni correction (p < 0.0125 (0.05/4) was the corrected statistically significant level in association analysis between the individual SNP and type 2 diabetes)

The major allele was used as a reference when comparing the risk of diabetes between alleles.

### Associations of *GCKR *polymorphisms with quantitative traits in non-diabetic controls

In the non-diabetic controls, the GG and AG carriers of rs3817588 were nominally associated with a lower plasma triglyceride level compared with the AA carriers after adjustment for year of birth, sex and BMI (p = 0.0003 and p = 0.02, respectively), and the trend was in the same direction (p for trend = 0.00004) (Table 4). The association of rs3817588 with plasma triglyceride level was still significant after correction for multiple testing. The GG carriers of rs780094 were nominally associated with a lower plasma triglyceride level compared with the AA carriers, after adjustment for year of birth, sex and BMI (p = 0.01), and the trend was in the same direction (p for trend = 0.03) (Table 4). However, the association of rs780094 with plasma triglyceride level was not significant after correction for multiple testing. The associations of rs8179206 and rs2293572 with plasma triglyceride level were not significant (Table 4). The GG carriers of rs3817588 were nominally associated with a higher waist circumference compared with the AA carriers, after adjustment for year of birth and sex (p = 0.01), and the trend was in the same direction (p for trend = 0.04) (Table 4). The association was not significant after correction for multiple testing. The associations of other polymorphisms with waist circumference were not significant (Table 4). None of the four polymorphisms showed a significant association with BMI, fasting plasma glucose, total cholesterol, HDL-C, LDL-C, systolic blood pressure or diastolic blood pressure (Table 4).

**Table 4:** Quantitative traits stratified according to *GCKR *genotypes in non-diabetic controls

	n	BMI (kg/m^2^)	Waist circumference (cm)	Fasting glucose (mmol/l)	Total cholesterol* (mmol/l)	Triglyceride* (mmol/l)	HDL-C* (mmol/l)	LDL-C* (mmol/l)	Systolic BP (mmHg)	Diastolic BP (mmHg)
rs8179206										
AA	1076	23.50 ± 0.09	80.50 ± 0.29	4.86 ± 0.01	5.11 ± 0.03	1.45 ± 0.02	1.32 ± 0.01	3.01 ± 0.03	119.14 ± 0.46	77.95 ± 0.27
AG	68	23.33 ± 0.39	81.48 ± 1.23	4.86 ± 0.05	5.04 ± 0.11	1.52 ± 0.09	1.30 ± 0.03	2.95 ± 0.10	122.28 ± 1.99	79.55 ± 1.11
GG^$^	2	19.47 ± /	68.00 ± /	4.10 ± /	3.80 ± /	0.90 ± /	1.16 ± /	2.23 ± /	110.00 ± /	70.00 ± /
P value^a#^		0.36	0.33	0.19	0.27	0.51	0.61	0.53	0.45	0.76
P value^b#^				0.15	0.31	0.49	0.24	0.64	0.35	0.72
rs2293572										
GG	842	23.53 ± 0.10	80.60 ± 0.32	4.85 ± 0.01	5.09 ± 0.03	1.44 ± 0.02	1.32 ± 0.01	3.00 ± 0.03	119.32 ± 0.53	78.13 ± 0.31
GC	276	23.37 ± 0.17	80.22 ± 0.58	4.87 ± 0.02	5.16 ± 0.06	1.51 ± 0.04	1.31 ± 0.02	3.03 ± 0.05	118.96 ± 0.90	77.54 ± 0.50
CC	23	24.03 ± 0.53	80.00 ± 1.85	4.98 ± 0.06	5.39 ± 0.19	1.53 ± 0.15	1.39 ± 0.06	3.12 ± 0.17	121.24 ± 1.95	78.96 ± 1.29
P value^a#^		0.48	0.63	0.18	0.17	0.28	0.54	0.74	0.14	0.24
P value^b#^				0.14	0.17	0.15	0.37	0.73	0.19	0.44
rs3817588										
AA	598	23.35 ± 0.12	79.88 ± 0.40	4.85 ± 0.02	5.13 ± 0.04	1.50 ± 0.03	1.32 ± 0.01	3.02 ± 0.04	119.27 ± 0.66	77.79 ± 0.38
AG	423	23.52 ± 0.15	81.14 ± 0.47	4.87 ± 0.02	5.08 ± 0.05	1.41 ± 0.03	1.33 ± 0.02	3.00 ± 0.04	118.93 ± 0.76	78.12 ± 0.45
GG	103	23.97 ± 0.30	82.13 ± 1.01	4.84 ± 0.04	4.99 ± 0.10	1.26 ± 0.05	1.32 ± 0.03	2.95 ± 0.08	119.58 ± 1.68	77.77 ± 0.94
P value^a#^		0.15	0.04	0.84	0.35	0.001^&^	0.78	0.82	0.59	0.76
P value^b#^				0.81	0.29	0.00004^&^	0.70	0.84	0.39	0.63
rs780094										
AA	368	23.22 ± 0.16	79.66 ± 0.50	4.84 ± 0.02	5.12 ± 0.05	1.49 ± 0.04	1.32 ± 0.02	3.02 ± 0.05	119.13 ± 0.87	77.83 ± 0.50
AG	558	23.59 ± 0.13	80.80 ± 0.42	4.86 ± 0.02	5.13 ± 0.04	1.44 ± 0.03	1.32 ± 0.01	3.02 ± 0.04	119.73 ± 0.70	78.40 ± 0.39
GG	218	23.67 ± 0.20	81.34 ± 0.70	4.85 ± 0.03	5.00 ± 0.07	1.35 ± 0.04	1.32 ± 0.02	2.92 ± 0.06	119.15 ± 1.04	77.33 ± 0.62
P value^a#^		0.15	0.16	0.74	0.32	0.06	0.79	0.40	0.96	0.24
P value^b#^				0.63	0.31	0.03	0.30	0.43	0.99	0.18

Data are expressed as the means ± SEM

^a ^Adjusted for year of birth and sex

^b ^Adjusted for year of birth, sex and BMI

* Variables were log-transformed before statistical analysis; numbers in the table were back-transformed

^# ^p for trend

^$ ^This group had too few subjects and no SEM was calculated

^&^Significant after Bonferroni correction (p < 0.0014 (0.05/36) was the corrected statistically significant level in association analysis between the individual SNP and quantitative traits in the control group)

### Associations of *GCKR *polymorphisms with AIR and ACPR in diabetic cases

**Figure 1:**
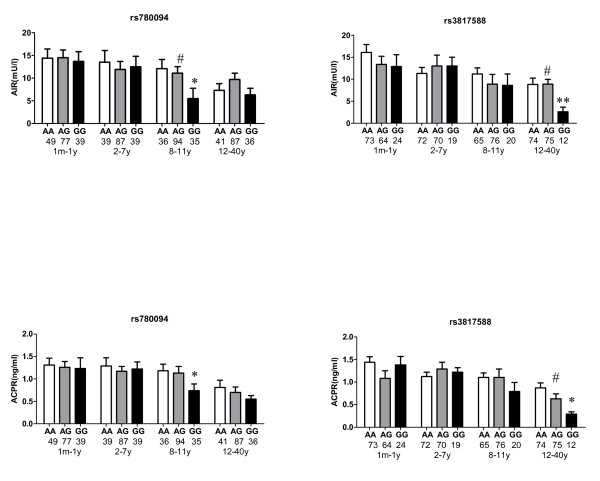
**Acute insulin response (AIR) and acute C-peptide response (ACPR) stratified according to *GCKR *rs780094 or rs3817588 genotypes by quartile of diabetic duration**. * p < 0.05 compared with homozygote of major allele (AA) ** p < 0.01 compared with homozygote of major allele (AA) # p for trend < 0.05 All p values were not significant after Bonferroni correction (p < 0.0016 (0.05/32) was used as Bonferroni corrected statistically significant level in association analysis between the individual SNP and AIR or ACPR) The figures below the genotypes indicate the number of diabetic cases for each genotype group.

A subgroup of diabetic cases (n = 664) was classified into 4 groups according to the duration of diabetes. The range of diabetes duration was 1 month to 40 years in this subgroup of diabetic patients. In the third quartile subgroup with a diabetic duration of 8-11 years, the GG carriers of rs780094 were nominally associated with lower levels of AIR and ACPR compared with the AA carriers, after adjustment for year of birth, sex and BMI (p = 0.02 and 0.03, respectively), and the trend was in the same direction (p for trend = 0.03 and 0.09, respectively) (Figure 1). In the fourth quartile subgroup with a diabetic duration over 11 years, the GG carriers of rs3817588 were nominally associated with lower levels of AIR and ACPR compared with the AA carriers, after adjustment for year of birth, sex and BMI (p = 0.008 and 0.01, respectively), and the trend was in the same direction (p for trend = 0.03 and 0.03, respectively) (Figure 1). However, these associations were no longer significant after correction for multiple testing.

### Associations of *GCKR *haplotypes with type 2 diabetes

The haplotype block was constructed for the four SNPs (rs8179206, rs2293572, rs3817588 and rs780094) in *GCKR*. All four SNPs fell into one block (Additional file 1, Figure S1). The block was associated with type 2 diabetes after adjustment for year of birth and sex (the global p value = 2.7 × 10^-5^) (Additional file 1, Table S1). The haplotypes AGGG and GGGG were associated with an increased risk of type 2 diabetes (OR = 1.18, 95% CI 1.02-1.37, p = 0.02 and OR = 2.08, 95% CI 1.60-2.71, p = 5.9 × 10^-8^, respectively) (Additional file 1, Table S1). The associations remained similar after additional adjustment for BMI (Additional file 1, Table S1).

### Associations of *GCKR *haplotypes with quantitative traits in non-diabetic controls

In the non-diabetic controls, the haplotype block was nominally associated with plasma triglyceride level adjusted for year of birth, sex and BMI (the global p value = 0.0001) (Additional file 1, Table S2). The haplotype AGGG was associated with a lower plasma triglyceride level adjusted for year of birth, sex and BMI (p = 2.2 × 10^-5^). The association remained significant after correction for multiple testing. BMI, waist circumference, fasting plasma glucose, total cholesterol, HDL-C, LDL-C, systolic blood pressure and diastolic blood pressure were not significantly different between haplotypes (Additional file 1, Table S2).

## Discussion

In line with previous studies, our study confirmed the opposite effects of *GCKR *variants on glucose and triglyceride concentrations. Our data showed that the *GCKR *rs780094 G allele and rs3817588 G allele were associated with an increased risk of type 2 diabetes in Han Chinese individuals. The G alleles were also nominally associated with a lower fasting triglyceride level. Moreover, the association of rs3817588 with fasting triglyceride level was still significant after correction for multiple testing. The associations of *GCKR *rs780094 with type 2 diabetes and triglyceride level have been replicated in many studies of different ethnic populations since the Diabetes Genetics Initiative genome-wide association study [7-11]. Our study confirmed this association again in a Han Chinese population. Our study was the first study which adopted a tagging strategy and selected tag SNPs of *GCKR *including 20 kb upstream and 9 kb downstream of the gene for studying. We demonstrated that another polymorphism rs3817588 in *GCKR *affected glucose and lipid metabolism in a similar way as rs780094, which was not reported in the previous studies.

In the present study, the G allele of *GCKR *rs780094 was associated with higher odds of type 2 diabetes (OR = 1.19). The effect size was similar to that observed in another study of a Han Chinese population [12]. In the studies of populations of European descent, the G allele of rs780094 was also associated with a higher odds of diabetes, but the effect size was much smaller (OR = 1.06-1.08) [8,10,11]. The frequency of the rs780094 G allele is substantially lower in Han Chinese (43%) than in White Europeans (65%). The difference in genetic background between different ethnic groups may explain the discrepancy between effect sizes in Han Chinese and European populations. The G allele of GCKR rs3817588 was associated with higher odds of type 2 diabetes (OR = 1.21). For both polymorphisms, the G risk alleles for diabetes were nominally associated with a lower triglyceride level. The mechanism by which the *GCKR *variants lead to type 2 diabetes and protect against dyslipidemia remains to be determined. A potential explanation is that GCK regulation by GCKR is altered in the liver, which leads indirectly to decreased GCK activity [17]. Decreased GCK activity was associated with decreased glucose utilization in the liver [17]. With decreased glucose utilization and glycolytic flux, GCK, phosphofructokinase, and fatty acid synthase are downregulated, whereas phosphoenolpyruvate carboxykinase and glucose-6-phosphatase are upregulated [17]. These changes increase hepatic glucose output, lower malonyl-CoA concentration and inhibit de novo lipogenesis and VLDL triglyceride production [17].

We next investigated whether the *GCKR *variants were associated with beta cell function as determined by an arginine stimulation test in diabetic patients. We found that in groups with a relatively long diabetic duration, *GCKR *variants were associated with AIR and ACPR. The carriers of the G alleles of rs780094 and rs3817588 had lower values for AIR and ACPR than the AA homozygotes. These findings suggested that the *GCKR *variants probably contribute to diabetes susceptibility by impairing beta cell function, although the associations of the *GCKR *variants with AIR and ACPR became non-significant after correction for multiple testing. A study in a Han Chinese population found that rs780094 was associated with beta cell function as estimated by HOMA-B, which was consistent with our findings [12].

We also did haplotype analysis and found that all four SNPs exhibited moderate to strong LD in terms of D' and fell into one block. The block was associated with type 2 diabetes after adjustment for year of birth, sex and BMI. The AGGG haplotype and the GGGG haplotype were associated with an increased risk of type 2 diabetes. Both haplotypes carried the G risk alleles of rs3817588 and rs780094. However, the ACAG haplotype which carried the G risk allele of rs780094 did not show any association with type 2 diabetes. This would suggest that the effect of the rs780094 G allele on the risk of type 2 diabetes was due to its LD with rs3817588. The difference of the AGGG haplotype and the GGGG haplotype was at the locus of rs8179206. However, the GGGG haplotype was associated with a 2.08 times risk of diabetes which was much higher than that of the AGGG haplotype (OR = 1.18), suggesting that the G alleles of rs8179206 and rs3817588 had synergistic effect on the development of type 2 diabetes. Rs8179206 G allele also contributed to the risk of type 2 diabetes although it was not associated with diabetes in single locus analysis.

Rs3817588 and rs780094 are located in introns of *GCKR*. Based on the current data, we cannot confirm whether they are in the splicing site or the transcription factor binding site of the gene. We therefore assume them to be linked with one or more functional variants within the *GCKR *gene or its regulatory regions. Rs780094 is tightly linked with rs1260326 (HapMap CEU r^2 ^= 0.93, CHB r^2 ^= 0.82), a non-synonymous variant in *GCKR *associated with type 2 diabetes and triglyceride level [9,10,12]. A functional study showed that *GCKR *rs1260326 was associated with fasting plasma glucose and triglyceride levels, and this effect was mediated through regulating the activity of GCK in liver [17]. We did not genotype rs1260326 in the current study because of the fact that it is in strong LD with rs780094 and represents the same information as rs780094, which was demonstrated by a previous study in a Han Chinese population [12]. Although there is no evidence that rs3817588 is linked with any functional variant now, future fine mapping and resequencing of the *GCKR *gene may detect such functional variants.

Our study had some limitations. Firstly, we did not investigate gene-environment interactions. Because both genetic variants and environmental factors contribute to type 2 diabetes, and adverse environmental factors (high-caloric diets, physical inactivity, etc.) have important influence on the development of diabetes, the elucidation of gene-environment interactions should not be overlooked in future studies. Secondly, we did not do a functional study of rs3817588. Further functional study is needed to determine whether rs3817588 is associated with selective splicing of mRNA or the binding of transcription factors and affects expression level of protein ultimately.

## Conclusions

We demonstrated that the rs3817588 A/G polymorphism of the *GCKR *gene was associated with type 2 diabetes and plasma triglyceride level in the Han Chinese population.

## Abbreviations

GCK: glucokinase; GCKR: glucokinase regulatory protein; HOMA-IR: homeostasis model assessment of insulin resistance; HOMA-B: homeostasis model assessment of beta cell function; IFG: impaired fasting glucose; AIR: acute insulin response; ACPR: acute C-peptide response; SNP: single nucleotide polymorphism; MAF: minor allele frequency; LD: linkage disequilibrium; SEM: standard error of mean; BMI: body mass index; CI: confidence interval; OR: odds ratio; HDL-C: high density lipoprotein cholesterol; LDL-C: low density lipoprotein cholesterol; BP: blood pressure; DM: diabetes; HWE: Hardy-Weinberg equilibrium.

## Competing interests

The authors declare that they have no competing interests.

## Authors' contributions

YL participated in the design of the study, carried out the SNP genotyping and the statistical analysis of the genotype data, and drafted the manuscript. XG contributed to the design and coordination of the study, to the statistical analysis, interpreted the findings and drafted the manuscript. XL, QG, HC and DL participated in the design of the study and the SNP genotyping. All authors read and approved the final manuscript.

## Pre-publication history

The pre-publication history for this paper can be accessed here:

http://www.biomedcentral.com/1471-2350/12/66/prepub

## Supplementary Material

Additional file 1 **Table S1 Association of *GCKR *haplotypes with type 2 diabetes**. Table S2 Quantitative traits stratified according to *GCKR *haplotypes in non-diabetic controls Figure S1 Haploview-generated linkage disequilibrium (LD) map and blocks of the 4 SNPs at the *GCKR *locus Click here for file

## References

[B1] Matschinsky FM Banting lecture 1995. A lesson in metabolic regulation inspired by the glucokinase glucose sensor paradigm Diabetes 1996 45 2 223 241 10.2337/diabetes.45.2.223 8549869

[B2] Van Schaftingen E A protein from rat liver confers to glucokinase the property of being antagonistically regulated by fructose 6-phosphate and fructose 1-phosphate Eur J Biochem 1989 179 1 179 184 10.1111/j.1432-1033.1989.tb14538.x 2917560

[B3] Chu CA Fujimoto Y Igawa K Grimsby J Grippo JF Magnuson MA Cherrington AD Shiota M Rapid translocation of hepatic glucokinase in response to intraduodenal glucose infusion and changes in plasma glucose and insulin in conscious rats Am J Physiol Gastrointest Liver Physiol 2004 286 4 G627 G634 10.1152/ajpgi.00218.2003 14656711

[B4] Farrelly D Brown KS Tieman A Ren J Lira SA Hagan D Gregg R Mookhtiar KA Hariharan N Mice mutant for glucokinase regulatory protein exhibit decreased liver glucokinase: a sequestration mechanism in metabolic regulation Proc Natl Acad Sci USA 1999 96 25 14511 14516 10.1073/pnas.96.25.14511 10588736 PMC24467

[B5] Grimsby J Coffey JW Dvorozniak MT Magram J Li G Matschinsky FM Shiota C Kaur S Magnuson MA Grippo JF Characterization of glucokinase regulatory protein-deficient mice J Biol Chem 2000 275 11 7826 7831 10.1074/jbc.275.11.7826 10713097

[B6] Slosberg ED Desai UJ Fanelli B St Denny I Connelly S Kaleko M Boettcher BR Caplan SL Treatment of type 2 diabetes by adenoviral-mediated overexpression of the glucokinase regulatory protein Diabetes 2001 50 8 1813 1820 10.2337/diabetes.50.8.1813 11473043

[B7] Diabetes Genetics Initiative of Broad Institute of Harvard and MIT, Lund University, and Novartis Institutes of BioMedical Research Saxena R Voight BF Lyssenko V Burtt NP de Bakker PI Chen H Roix JJ Kathiresan S Hirschhorn JN Daly MJ Hughes TE Groop L Altshuler D Almgren P Florez JC Meyer J Ardlie K Bengtsson Boström K Isomaa B Lettre G Lindblad U Lyon HN Melander O Newton-Cheh C Nilsson P Orho-Melander M Råstam L Speliotes EK Taskinen MR Tuomi T Guiducci C Berglund A Carlson J Gianniny L Hackett R Hall L Holmkvist J Laurila E Sjögren M Sterner M Surti A Svensson M Svensson M Tewhey R Blumenstiel B Parkin M Defelice M Barry R Brodeur W Camarata J Chia N Fava M Gibbons J Handsaker B Healy C Nguyen K Gates C Sougnez C Gage D Nizzari M Gabriel SB Chirn GW Ma Q Parikh H Richardson D Ricke D Purcell S Genome-wide association analysis identifies loci for type 2 diabetes and triglyceride levels Science 2007 316 5829 1331 1336 17463246 10.1126/science.1142358

[B8] Sparsø T Andersen G Nielsen T Burgdorf KS Gjesing AP Nielsen AL Albrechtsen A Rasmussen SS Jørgensen T Borch-Johnsen K Sandbaek A Lauritzen T Madsbad S Hansen T Pedersen O The GCKR rs780094 polymorphism is associated with elevated fasting serum triacylglycerol, reduced fasting and OGTT-related insulinaemia, and reduced risk of type 2 diabetes Diabetologia 2008 51 1 70 75 18008060 10.1007/s00125-007-0865-z

[B9] Vaxillaire M Cavalcanti-Proença C Dechaume A Tichet J Marre M Balkau B Froguel P DESIR Study Group: The common P446L polymorphism in GCKR inversely modulates fasting glucose and triglyceride levels and reduces type 2 diabetes risk in the DESIR prospective general French population Diabetes 2008 57 8 2253 2257 10.2337/db07-1807 18556336 PMC2494697

[B10] Orho-Melander M Melander O Guiducci C Perez-Martinez P Corella D Roos C Tewhey R Rieder MJ Hall J Abecasis G Tai ES Welch C Arnett DK Lyssenko V Lindholm E Saxena R de Bakker PI Burtt N Voight BF Hirschhorn JN Tucker KL Hedner T Tuomi T Isomaa B Eriksson KF Taskinen MR Wahlstrand B Hughes TE Parnell LD Lai CQ Berglund G Peltonen L Vartiainen E Jousilahti P Havulinna AS Salomaa V Nilsson P Groop L Altshuler D Ordovas JM Kathiresan S Common missense variant in the glucokinase regulatory protein gene is associated with increased plasma triglyceride and C-reactive protein but lower fasting glucose concentrations Diabetes 2008 57 3112 3121 10.2337/db08-0516 18678614 PMC2570409

[B11] Dupuis J Langenberg C Prokopenko I Saxena R Soranzo N Jackson AU Wheeler E Glazer NL Bouatia-Naji N Gloyn AL Lindgren CM Mägi R Morris AP Randall J Johnson T Elliott P Rybin D Thorleifsson G Steinthorsdottir V Henneman P Grallert H Dehghan A Hottenga JJ Franklin CS Navarro P Song K Goel A Perry JR Egan JM Lajunen T Grarup N Sparsø T Doney A Voight BF Stringham HM Li M Kanoni S Shrader P Cavalcanti-Proença C Kumari M Qi L Timpson NJ Gieger C Zabena C Rocheleau G Ingelsson E An P O'Connell J Luan J Elliott A McCarroll SA Payne F Roccasecca RM Pattou F Sethupathy P Ardlie K Ariyurek Y Balkau B Barter P Beilby JP Ben-Shlomo Y Benediktsson R Bennett AJ Bergmann S Bochud M Boerwinkle E Bonnefond A Bonnycastle LL Borch-Johnsen K Böttcher Y Brunner E Bumpstead SJ Charpentier G Chen YD Chines P Clarke R Coin LJ Cooper MN Cornelis M Crawford G Crisponi L Day IN de Geus EJ Delplanque J Dina C Erdos MR Fedson AC Fischer-Rosinsky A Forouhi NG Fox CS Frants R Franzosi MG Galan P Goodarzi MO Graessler J Groves CJ Grundy S Gwilliam R Gyllensten U Hadjadj S Hallmans G Hammond N Han X Hartikainen AL Hassanali N Hayward C Heath SC Hercberg S Herder C Hicks AA Hillman DR Hingorani AD Hofman A Hui J Hung J Isomaa B Johnson PR Jørgensen T Jula A Kaakinen M Kaprio J Kesaniemi YA Kivimaki M Knight B Koskinen S Kovacs P Kyvik KO Lathrop GM Lawlor DA Le Bacquer O Lecoeur C Li Y Lyssenko V Mahley R Mangino M Manning AK Martínez-Larrad MT McAteer JB McCulloch LJ McPherson R Meisinger C Melzer D Meyre D Mitchell BD Morken MA Mukherjee S Naitza S Narisu N Neville MJ Oostra BA Orrù M Pakyz R Palmer CN Paolisso G Pattaro C Pearson D Peden JF Pedersen NL Perola M Pfeiffer AF Pichler I Polasek O Posthuma D Potter SC Pouta A Province MA Psaty BM Rathmann W Rayner NW Rice K Ripatti S Rivadeneira F Roden M Rolandsson O Sandbaek A Sandhu M Sanna S Sayer AA Scheet P Scott LJ Seedorf U Sharp SJ Shields B Sigurethsson G Sijbrands EJ Silveira A Simpson L Singleton A Smith NL Sovio U Swift A Syddall H Syvänen AC Tanaka T Thorand B Tichet J Tönjes A Tuomi T Uitterlinden AG van Dijk KW van Hoek M Varma D Visvikis-Siest S Vitart V Vogelzangs N Waeber G Wagner PJ Walley A Walters GB Ward KL Watkins H Weedon MN Wild SH Willemsen G Witteman JC Yarnell JW Zeggini E Zelenika D Zethelius B Zhai G Zhao JH Zillikens MC DIAGRAM Consortium GIANT Consortium Global BPgen Consortium Borecki IB Loos RJ Meneton P Magnusson PK Nathan DM Williams GH Hattersley AT Silander K Salomaa V Smith GD Bornstein SR Schwarz P Spranger J Karpe F Shuldiner AR Cooper C Dedoussis GV Serrano-Ríos M Morris AD Lind L Palmer LJ Hu FB Franks PW Ebrahim S Marmot M Kao WH Pankow JS Sampson MJ Kuusisto J Laakso M Hansen T Pedersen O Pramstaller PP Wichmann HE Illig T Rudan I Wright AF Stumvoll M Campbell H Wilson JF Anders Hamsten on behalf of Procardis Consortium MAGIC investigators Bergman RN Buchanan TA Collins FS Mohlke KL Tuomilehto J Valle TT Altshuler D Rotter JI Siscovick DS Penninx BW Boomsma DI Deloukas P Spector TD Frayling TM Ferrucci L Kong A Thorsteinsdottir U Stefansson K van Duijn CM Aulchenko YS Cao A Scuteri A Schlessinger D Uda M Ruokonen A Jarvelin MR Waterworth DM Vollenweider P Peltonen L Mooser V Abecasis GR Wareham NJ Sladek R Froguel P Watanabe RM Meigs JB Groop L Boehnke M McCarthy MI Florez JC Barroso I New genetic loci implicated in fasting glucose homeostasis and their impact on type 2 diabetes risk Nature Genetics 2010 42 2 105 116 10.1038/ng.520 20081858 PMC3018764

[B12] Qi Q Wu Y Li H Loos RJ Hu FB Sun L Lu L Pan A Liu C Wu H Chen L Yu Z Lin X Association of GCKR rs780094, alone or in combination with GCK rs1799884, with type 2 diabetes and related traits in a Han Chinese population Diabetologia 2009 52 5 834 843 10.1007/s00125-009-1290-2 19241058

[B13] Tam CH Ma RC So WY Wang Y Lam VK Germer S Martin M Chan JC Ng MC Interaction effect of genetic polymorphisms in glucokinase (GCK) and glucokinase regulatory protein (GCKR) on metabolic traits in healthy Chinese adults and adolescents Diabetes 2009 58 3 765 769 19073768 10.2337/db08-1277 PMC2646078

[B14] Wen J Rönn T Olsson A Yang Z Lu B Du Y Groop L Ling C Hu R Investigation of type 2 diabetes risk alleles support CDKN2A/B, CDKAL1, and TCF7L2 as susceptibility genes in a Han Chinese cohort PLoS One 2010 5 2 e9153 10.1371/journal.pone.0009153 20161779 PMC2818850

[B15] World Health Organization Definition, diagnosis and classification of diabetes mellitus and its complication WHO/NCD/NCS 1999 31 32

[B16] Carlson CS Eberle MA Rieder MJ Yi Q Kruglyak L Nickerson DA Selecting a maximally informative set of single-nucleotide polymorphisms for association analyses using linkage disequilibrium Am J Hum Genet 2004 74 1 106 120 10.1086/381000 14681826 PMC1181897

[B17] Beer NL Tribble ND McCulloch LJ Roos C Johnson PR Orho-Melander M Gloyn AL The P446L variant in GCKR associated with fasting plasma glucose and triglyceride levels exerts its effect through increased glucokinase activity in liver Hum Mol Genet 2009 18 21 4081 4088 10.1093/hmg/ddp357 19643913 PMC2758140

