# Supplementary material for: Associations of common polymorphisms in GCKR with type 2 diabetes and related traits in a Han Chinese population: a case-control study
Source: BMC Med Genet. 2011 May 13;12:66. doi: 10.1186/1471-2350-12-66 (PMC3112072; doi:10.1186/1471-2350-12-66)
Supplement: Additional file 1 — Table S1 Association of GCKR haplotypes with type 2 diabetes. Table S2 Quantitative traits stratified according to GCKR haplotypes in non-diabetic controls Figure S1 Haploview-generated linkage disequilibrium (LD) map and blocks of the 4 SNPs at the GCKR locus [file 1471-2350-12-66-S1.DOC]

**Figure S1: Haploview-generated linkage disequilibrium (LD) map and blocks of the 4 SNPs at the *GCKR* locus**

D' (*100) was showed on each rectangle (D' is a statistical measure of linkage disequilibrium, D' = 1 is known as complete linkage, and the darker of the color the higher of the linkage).

**Table S1: Association of *GCKR* haplotypes with type 2 diabetes**

|  | Haplotype | Non-DM  (%) | DM  (%) | Odds Ratio1^a^ | 95% CI | P value | Odds Ratio2^b^ | 95% CI | P value |
| --- | --- | --- | --- | --- | --- | --- | --- | --- | --- |
| H1 | AGAA | 56.3 | 51.6 | 1.00 | (Ref.) | 2.7×10^-5^* | 1.00 | (Ref.) | 6.0×10^-6^* |
| H2 | AGGG | 25.5 | 27.7 | 1.18 | 1.02-1.37 | 0.02 | 1.21 | 1.04-1.41 | 0.01 |
| H3 | ACAG | 13.3 | 12.0 | 1.01 | 0.83-1.23 | 0.91 | 1.04 | 0.85-1.27 | 0.73 |
| H4 | GGGG | 4.9 | 8.6 | 2.08 | 1.60-2.71 | 5.9×10^-8^ | 2.22 | 1.68-2.94 | 1.9×10^-8^ |

^a^ Adjusted for year of birth and sex

^b^ Adjusted for year of birth, sex and BMI

* Global p value

The most frequent haplotype was used as a reference when comparing the risk of diabetes between different haplotypes

**Table S2: Quantitative traits stratified according to *GCKR* haplotypes in non-diabetic controls**

| Haplotype | BMI  (kg/m^2^) | Waist circumference  (cm) | Fasting glucose  (mmol/l) | Total cholesterol*  (mmol/l) | Triglyceride*  (mmol/l) | HDL-C*  (mmol/l) | LDL-C*  (mmol/l) | Systolic BP  (mmHg) | Diastolic BP  (mmHg) |
| --- | --- | --- | --- | --- | --- | --- | --- | --- | --- |
| AGAA | 23.38±0.08 | 80.15±0.28 | 4.85±0.01 | 5.14±0.03 | 1.47±0.02 | 1.32±0.01 | 3.04±0.03 | 119.33±0.47 | 78.09±0.27 |
| AGGG | 23.72±0.13 | 81.49±0.42 | 4.86±0.02 | 5.08±0.04 | 1.34±0.03 | 1.34±0.03 | 3.03±0.03 | 118.73±0.68 | 77.84±0.40 |
| ACAG | 23.44±0.17 | 80.15±0.60 | 4.89±0.02 | 5.18±0.06 | 1.49±0.04 | 1.32±0.02 | 3.03±0.05 | 119.25±0.87 | 77.47±0.49 |
| GGGG | 23.51±0.30 | 81.06±0.98 | 4.80±0.04 | 4.94±0.10 | 1.45±0.07 | 1.31±0.03 | 2.85±0.08 | 121.00±1.67 | 78.85±0.92 |
| p value^a#^  p value^b#^ | 0.24 | 0.09 | 0.17 | 0.10 | 0.001^$^ | 0.87 | 0.21 | 0.44 | 0.51 |
|  |  |  | 0.19 | 0.18 | 0.0001^$^ | 0.71 | 0.42 | 0.35 | 0.36 |

Data are expressed as means ± SEM

^a^ Adjusted for year of birth and sex

^b^ Adjusted for year of birth, sex and BMI

* Variables were log-transformed before statistical analysis; numbers in the table were back-transformed

^#^ Global p value

^$^ Significant after Bonferroni correction (p < 0.0056 (0.05/9) was the corrected statistically significant level in association analysis between haplotype and quantitative traits in the control group)

*

*

Untranslated region

Intron

Chromosome2; Location: 2p23.3

Exon

3’”””

5’”””


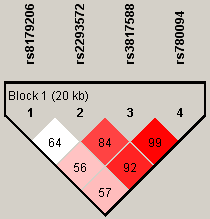


**Figure S1**
